# Quantum Contextuality and Indeterminacy

**DOI:** 10.3390/e22080867

**Published:** 2020-08-07

**Authors:** Gregg Jaeger

**Affiliations:** Quantum Communication and Measurement Laboratory, Department of Electrical and Computer Engineering, and Division of Natural Science and Mathematics, Boston University, Boston, MA 02215, USA; jaeger@bu.edu

**Keywords:** contextuality, uncertainty relations, indeterminacy relations, unsharp observable, unsharp reality, localization, POVM, quantum mechanics, Copenhagen interpretation

## Abstract

The circumstances of measurement have more direct significance in quantum than in classical physics, where they can be neglected for well-performed measurements. In quantum mechanics, the dispositions of the measuring apparatus-plus-environment of the system measured for a property are a non-trivial part of its formalization as the quantum observable. A straightforward formalization of context, via equivalence classes of measurements corresponding to sets of sharp target observables, was recently given for sharp quantum observables. Here, we show that quantum contextuality, the dependence of measurement outcomes on circumstances external to the measured quantum system, can be manifested not only as the strict exclusivity of different measurements of sharp observables or valuations but via quantitative differences in the property statistics across simultaneous measurements of *generalized* quantum observables, by formalizing quantum context via coexistent generalized observables rather than only its subset of compatible sharp observables. Here, the question of whether such quantum contextuality follows from basic quantum principles is then addressed, and it is shown that the Principle of Indeterminacy is sufficient for at least one form of non-trivial contextuality. Contextuality is thus seen to be a natural feature of quantum mechanics rather than something arising only from the consideration of impossible measurements, abstract philosophical issues, hidden-variables theories, or other alternative, classical models of quantum behavior.

## 1. Introduction

The dependence of measurement results on their physical circumstances, known as quantum contextuality, and its extension beyond quantum mechanics have been of increased interest in the foundations of quantum theory and elsewhere where measurement and information may play an important role, including linguistics; cf., for example, References [1,2,3,4,5]. The first notion of contextuality in quantum mechanics arose from the requirement of specifying the circumstances of measurements of quantities insisted on by Niels Bohr and Werner Heisenberg, who noted the possible dependence and, most strikingly, nonexistence of the outcome of any non-trivial measurement due to other measurements performed or performable in principle on the measured system [1]; the specification of such circumstances serves, de facto, as the specification of what we here consider quantum context.

Possible hidden-variables supplementing observables, considered in the 1930s by Heisenberg, and arguments of John von Neumann against such variables have since informed explorations of contextuality in and, especially, beyond quantum mechanics, but appear also to have obscured its natural quantum character. A form of context natural to quantum mechanics is definable in terms of sets of quantum observables and their characteristics. Contextuality, defined as the variability of the statistics of measurement of a property across quantum contexts, has been considered in those terms: Context was recently defined as the equivalence class of experimental arrangements corresponding to a set of measurements of compatible quantum observables *O* represented by mutually commuting Hermitian operators which correspond to projection-valued measures (PVMs) and, so, to a set of such observables [1]. This is a practical definition of context for sharp measurements, in that it does not involve sets of observables that cannot be carried out simultaneously.

Here, a more general but still practical definition of context C is considered and defined as the equivalence class of experimental arrangements corresponding to a set of measurements of *coexistent* generalized observables, that is, a set of positive operator-valued measures *E* (POVMs) which can be obtained as marginals of a single joint POVM; cf. References [6,7,8,9]. This allows the significance of context to be quantitatively assessed rather than being viewed only as the manifestation of strict complementarity. This class of arrangements is then used to demonstrate that quantum contextuality can be grounded in the Principle of Indeterminacy, which is also amenable to quantitative expression, showing that contextuality is a genuine, natural, quantitative feature of quantum mechanics rather than being inherently qualitative or tied to hidden-variables theories or other alternative models of quantum behavior.

## 2. Background: Indeterminacy as Principle

The indeterminacy first identified in quantum mechanics was most broadly expressed by Heisenberg as the proposition that “canonically conjugate quantities can be determined simultaneously only with a characteristic inaccuracy” (*Ungenauigkeit*), a statement about imprecision of value of mechanical properties. Although the imprecision can be related to uncertainty, it does not primarily regard a lack of knowledge [10], but regards it only secondarily. Heisenberg later recalled that, while attempting to come to grips with this indeterminacy, Bohr “preferred to play between the particle- and wave-pictures while I tried to use the mathematical scheme and its probabilistic interpretation” which provides the statistics for properties in the allowed system states. Determination is generally taken to be concomitant with strict predicability following from the existence of laws underwriting a strict form of causation. The essence of Heisenberg’s quantum indeterminism is the idea that the precision of simultaneous specifiability of values of some *pairs* of physical magnitudes can be limited *as a matter of principle*, which then fundamentally *limits* predictability; cf., for example, Reference [11]. The extent of those fundamental limitations—in particular, those on the statistics of the quantities symbolized by what have come to be known as sharp quantum observables—were then formulated by him and others.

Heisenberg began by considering limitations on position and momentum in the formalism of quantum mechanics in light of the question, “Is it perhaps true that only such situations occur in nature or in experiments which can be represented in the mathematical scheme of quantum mechanics? That meant: there was not a real path of the electron in the cloud chamber. There was a sequence of water droplets. Each droplet determined inaccurately the position of the electron, and the velocity could be determined inaccurately from the sequence of droplets. “…the calculation gave a lower limit for the product of the inaccuracies of position and momentum. It remained to be demonstrated that the result of any well defined observation would obey this relation of uncertainty. Many experiments were discussed … The results confirmed the validity of the relations … that the way in which quantum theory was used in the analysis of observations, was compatible with the mathematical scheme…” [12], p. 5. Ultimately, Heisenberg concluded that “in the sharp formulation of the law of causality, ‘If we know the present exactly, we can calculate the future,’ it is not the consequent that is wrong, but the antecedent. We cannot in principle get to know the present in all determining data” ([13], p. 197). Such inherent unsharpness, considered here, has only relatively recently been well formalized in general, similarly to the initially formalized, sharp quantities [6].

The fundamental imprecision of quantities appearing in quantum mechanics contradicts the classical Principle of Determinacy attributed to Isaac Newton, that is, the thesis that the values of physical properties—for him, the positions and velocities of all the particles in the world—at some initial instant, in the absence of interventions, determine all their future and their past values; cf., for example, Reference [14], p. 68. The quantum limitation of physical specification was quickly considered a principle, and became known as the (Heisenberg) Principle of Indeterminacy (HPI), or Uncertainty Principle that suggested to him a specific interpretation of quantum mechanics that came to be associated with Copenhagen; cf. Reference [15]. For Heisenberg, “The main point in this new interpretation of quantum theory was the limitation in the applicability of the classical concepts. This limitation is in fact general and well defined; it applies to …position, velocity, energy, as well as amplitude, wavelength, density” ([12], pp. 5–6), which are considered intuitive (*anschaulich*).

## 3. Background: Indeterminacy Relations

The identified trade-off between the precision of simultaneous specification of position and of momentum, which Heisenberg discovered through the analysis of state measurement, was taken as an immediately generalizable example with which to infer both a principle and corresponding precise mathematical relations. The first instance of the expressions at which he arrived was

(1) $$q_{1}p_{1}∼h\;,$$

where $q_{1}=\delta q,p_{1}=\delta p$ correspond to measurement accuracies, but it was immediately generalized beyond this pair of conjugate properties in the same paper [13]. Heisenberg also gave the relation for simultaneous measurement (in)accuracies Δx,Δp of position and momentum as the inequality

(2) $$\Delta p_{x}\Delta x\geq h\;,$$

where $p_{x}$ indicates the component of momentum in the spatial *x*-direction [10], p. 14.

To arrive at these relations, Heisenberg had specifically considered as his archetype an experimental situation involving the optical probing of an electron. “At the instant of time when the position is determined, that is, at the instant when the photon is scattered by the electron, the electron undergoes a discontinuous change in momentum. This change is the greater the smaller the wavelength of the light employed, that is, the more exact the determination of the position. At the instant at which the position of the electron is known, its momentum therefore can be known only up to magnitudes which correspond to that discontinuous change;” [13], pp. 174–175.

Heisenberg arrived at the mathematical expression of his new principle after considering this together with Compton scattering within his theory of matrix mechanics, arguing “Thus, the more precisely the position is determined, the less precisely the momentum is known, and conversely. In this circumstance, we see a direct physical interpretation of the equation

(3) QP−PQ=iℏ,"

namely, that the measurement inaccuracies “stand in the relation” of Equation (1), which is “a straightforward mathematical consequence of the rule" of non-commutativity, Equation (3). He argued that this move also provides a demonstration of the *Anschaulichkeit* of this matrix (operator) relation; cf., for example, References [16,17]. This understanding of the limitations on theoretical notions was viewed as reinforced purely empirically (*rein erfahrungsgemäß*), in that experiments measuring pairs of such quantities are found to have such indeterminacies precluding their joint sharp observation.

These relations were immediately generalized to include time-energy and action-angle relations analogous to those for the position-momentum pair, by exploiting corresponding commutation relations and contemplating the methods for their observation. “The experiments which provide a [quantum] definition themselves suffer an indeterminacy introduced purely by the observational procedures we use when we ask of them the simultaneous determination of two canonically conjugate quantities. The magnitude of this indeterminacy is given by relation (1) (generalized to any canonically conjugate quantities whatsoever)” [13]. Heisenberg also noted that the indeterminacy relation (1) “can be derived from the Dirac-Jordan formulation by a slight generalization” involving translating quantum probabilities with corresponding Gaussian spreads, whereby one arrives at saturated versions of inequalities like Equation (2), for example, the equality

(4) $$\delta q_{1}\delta p_{1}=\hbar ,$$

that is, Equation (6) of Reference [13].

Quantum mechanics was soon more fully formalized in work of Paul Dirac [18] and von Neumann [19,20,21], wherein Heisenberg’s relations can be expressed in terms of the variances of Hermitian operators on Hilbert-space evaluated for particular quantum states, ρ. The variance (dispersion) of an operator *A*, given in a general quantum state, is ${\mathrm{Var}}_{\rho }A\equiv {\langle {\left(A-\langle A\rangle I\right)}^{2}\rangle }_{\rho }={\langle A^{2}\rangle }_{\rho }-{\langle A\rangle }_{\rho }^{2}.$ The square root of the variance, the standard deviation, is the ‘uncertainty’ $\Delta A\equiv \sqrt{{\mathrm{Var}}_{\rho }A}$ of *A* in state ρ; in the three-dimensional setting, Equation (2) is

(5) $$\Delta P_{i}\Delta Q_{i}\geq \frac{\hbar }{2}\delta_{ij},$$

where the indices are spatial components of these vectorial quantities for a massive particle such as the electron. In the general case, as Robertson showed shortly thereafter, “The uncertainty principle for two [Hermitian operators] whose commutator AB−BA=hC/2πi is

(6) $$\Delta A\Delta B\geq h|C_{0}|/4\pi ,"$$

where the values of the constants “if the two variables are conjugate, for then *C*, and consequently $C_{0}$ are ±1.” [22]. In the case of position and momentum, one has (in the modern formalism) then for the product of their variances

(7) $${\langle {\left(\Delta x\right)}^{2}\rangle }_{\rho }{\langle {\left(\Delta p_{x}\right)}^{2}\rangle }_{\rho }\geq \hbar^{2}/4.$$

It is possible to axiomatize quantum mechanics consistently with the approach of the above-mentioned workers by taking the set of observables *O* represented by Hermitian operators on complex Hilbert space H, that is, *sharp* observables, as basic axiomatic elements; cf. Reference [23], pp. 248–249. In this axiomatic approach, the state ψ of a quantum system can be considered a complete description of it; cf. Reference [23], pp. 248–249. The state is determined by the set of expectation values $\left\{s\left(A_{i}\right)\right\}$ for all $A_{i}\in O$, which are always well defined so long as the observables of *O* are bounded. Expectation values are required to be such that s(I)=1, where I is the identity operator, and that s(αA+βB)=αs(A)+βs(B)), for A,B∈O, for all real α,β, whenever *A* and *B* commute, with the additional imposition of a continuity condition, namely, that a sequence of bounded operators $A_{i}$ converges strongly to a bounded operator *A* if $A_{i}\psi \to A\psi$ for all vectors ψ∈H of the observables when prepared in it, that is, via the expectation functionals S on O. Then, for any two quantum (sharp) observables *A* and *B* and a generic state ρ, the general indeterminacy relation

(8) $${\langle {\left(\Delta A\right)}^{2}\rangle }_{\rho }{\langle {\left(\Delta B\right)}^{2}\rangle }_{\rho }\geq \frac{1}{4}{\left|{\langle \left[A,B\right]\rangle }_{\rho }\right|}^{2},$$

which is now referred to as the Heisenberg–Robertson relation, also follows Reference [22]. (For a straightforward contemporary derivation of this expression in bra-ket notation, see Reference [24], pp. 35–36.) Sharp observables of two canonically conjugate quantities, which provide a simple example of contextuality, discussed further in the next section, do not commute and the right hand side is non-zero as, for example, Equation (5) for i=j, and their joint measurement is impossible.

HPI, given in precise form such as Equation (6) or (8), expresses quantitively the in-principle limitations on the precision of joint determination of physical quantities, whatever might be the means of their measurement, and is connected with ψ as part of Heisenberg’s answer to the question “How can we translate the result of an observation into the mathematical scheme” incorporating Equation (3). This is the manner in which the Newtonian Principle of Determinacy is contradicted by HPI. A contemporary discussion of uncertainty relations in regard to measurement noise and disturbance can be found in Reference [25]. One may note that Heisenberg considered light in the form of a gamma-ray pulse as a tool for determining the position and momentum of an electron, but in his initial discussions pertinent here [10,13], his subject of interest was the question of the joint measurement of the properties of an electron rather than of the light pulse itself; however, uncertainty relations relating to light were to become of great interest later—a detailed discussion of its application in relation to photons and coherent states that involves the modern mathematical techniques of POVMs used here can be found in Reference [8].

## 4. Contextuality from Indeterminacy

With the background, origins, and senses of the HPI now in mind, our main result—that a form of quantitatively assessible contextuality is implied by the quantitative formulations of HPI—can be demonstrated which ultimately follows from the quantum characteristic that measurement at the atomic scale is such that “individual atomic processes…, due to their very nature, are determined by the interaction between the objects in question and the measurement instruments necessary for the definition of the experimental arrangement” [26]. This characteristic was to some extent indicated early on by the well-established results of von Neumann regarding the joint measurability of sharp observables. Recall that a set of measurements represented by commuting sharp observables, that is, Hermitian Hilbert-space operators *A* and *B* is *compatible* if $\left[A,B\right]=\hat{0}$ and *incompatible* if $\left[A,B\right]\neq \hat{0}$, that is, if two observables commute then they can be simultaneously measured, and *cannot be* if they do not commute; cf. Reference [19,20,21], Section III.3.

However, measurability per se is not part of the standard axiomatics of quantum theory nor of its general principles which are, by constrast, algebraic and statistical. (However, Schwinger’s abductive represents a sensible approach for going about this; cf. Reference [27], wherein the word “abduction" was changed to “induction” in typesetting, in error.) Because quantitative expressions of HPI, which is a general principle following from (at least one of) its axiomatization(s) and arrived at by Heisenberg from consideration of the statistics of measurements of the kind suggested by Equation (1), it is the natural place to locate the origin of the corresponding form of quantum contextuality.

Let us, therefore, define a (general) *quantum context*, C, as an equivalence class of physical arrangements for the measurement of a set of properties, symbolized by a set of observables, whether sharp *or* unsharp, where the statistics of outcomes of *coexistent* joint measurements of these observables are unaffected by the measurement of the others of that set. The definition of context provided here is a straightforward generalization of that given in Reference [1], which considered only sharp observables, that also involves the generalization from compatible to *coexistent* observables. The general class of observables includes both the traditional sharp observables as well as the other, unsharp observables. The maximally specified state of a quantum system relative to an observable *O* can be given as a projector, corresponding to a projection-valued measure, $\rho_{\mathrm{pure}}\equiv P_{o}=\left|\psi \rangle \langle \psi \right|$ appearing in the spectral decomposition of an observable *O*; cf. Reference [6], Section 2. Those observables representable as projectors are the *sharp* observables.

In addition to measurements corresponding to such operators, *unsharp observables* are the class of quantum operations that are described by (normalized) *positive-operator-valued measures*, POVMs; the POVMs are the natural correspondents in the operator space of quantum mechanics of standard probability measures and are defined as follows. Given a nonempty set *S* and a σ-algebra Σ of its subsets $X_{m}$, a POVM *E* is a collection of operators $\left\{E\left(X_{m}\right)\right\}$ satisfying three conditions: (i) *Positivity*—$E\left(X_{m}\right)\geq E\left(Ø\right)$, for all $X_{m}\in \Sigma$; (ii) *Additivity*—for all countable sequences of disjoint sets $X_{m}$ in Σ, $E\left(\cup_{m}X_{m}\right)=\sum_{m}E\left(X_{m}\right)$; (iii) *Completeness*—E(S)=I. If the *value space* (S,Σ) of a POVM *E* is a subspace of the real Borel space (R,B(R)), then *E* provides a unique Hermitian operator on H, namely $\int_{R}\mathrm{Id}\;dE,$ where Id is the identity map. It is the unsharp observables that allow us to consider contextuality more broadly as we do here.

The positive operators $E(X_{m})$ in the range of a POVM are referred to as *effects* E(H)={A∈L(H):O≤A≤I}, the expectation values of which provide the quantum probabilities. Given an effect *A*, *properties* are definable by the following set of conditions. (i) There exists a property $A^{⊥}$, where $A^{⊥}=I-A$ is the orthocomplementation of *A*; (ii) There exist states ρ and $\rho^{\prime }$ such that both $\mathrm{tr}\left(A\rho \right)>\frac{1}{2}$ and $\mathrm{tr}\left(A\rho^{\prime }\right)>\frac{1}{2}$; (iii) If *A* is regular, for any effect *B* below *A* and $A^{⊥}$, $2B\leq A+A^{⊥}=I$, where a *regular effect* is an effect with spectrum both above and below $\frac{1}{2}$. Thus, the set of properties is $E_{p}\left(H\right)=\left\{A\in E\left(H\right)|A≰\frac{1}{2}I,A≱\frac{1}{2}I\right\}\cup \left\{O,I\right\}$; the set of *unsharp properties* is $E_{u}\left(H\right)\equiv E{\left(H\right)}_{p}/L\left(H\right).$ A POVM is an *unsharp observable* if there exists an unsharp property in its range [7].

The probability of a given outcome *m* upon a (generalized) measurement on a system in a pure state P(|ψ〉) is

(9) $$p\left(m\right)=\langle \psi |E\left(X_{m}\right)|\psi \rangle =\mathrm{tr}\left((|\psi \rangle \langle \psi |)E\left(X_{m}\right)\right);$$

cf. Equation (1), which holds for the case of sharp measurement. The effects form a convex subset of the space of linear operators on L(H) on the system Hilbert space, the extremal elements of this subset being the projectors $\left\{P_{i}\right\}$ which, again, are those corresponding to the sharp observables.

A collection of effects is *coexistent* if the union of their ranges is contained within the range of a POVM. Any two quantum observables $E_{1}$ and $E_{2}$ are representable as sharp measures on (R,B(R)) exactly when $[E_{1},E_{2}]=O$, following from Reference [19,20,21]. Coexistent observables are thus those that can be *measured simultaneously in a common measurement arrangement*, and when two observables are coexistent, there exists an observable the statistics of which contain those of both observables, the *joint observable*. Typically, the two observables are recoverable as marginals of a joint distribution on the product of the corresponding two outcome spaces.

Although the fullest measurement of state possible can be given by a complete set of commuting operators, corresponding to a context $\left\{E_{1},E_{2},\ldots \right\}$ [1], the measurement of a set of coexistent unsharp observables may provide equally good state specifications, that is, be informationally complete; the latter are generalized observables corresponding to unsharp measurements which can be performed of two properties whose sharp observables are incompatible. Thus, the $E_{i}$ of a context are required here to be coexistent rather than commuting, as befits comeasurability for generalized observables. Along the lines similar to Reference [4], let us then take *contextuality* to be defined as follows. If a measurement of two or more properties is such that, for any system state, the joint measurement of two of them, AB, does not provide the same measurement statistics for A and B as those found when they are measured separately, then contextuality is present. This contextuality is readily found in the case of sharp observables. For example, consider the following quantities: position and momentum along the x-direction. Each quantity has a corresponding sharp observable, position *X* and momentum $P_{X}$, respectively, and, *when measured separately* (in different contexts), these quantities are each found to be determined as precisely as desired. When a system, say, an electron, prepared in the same state, say, centered on the origin of coordinates, separate measurements of these quantities in the two contexts $C_{1}=\left\{P_{X},Y\right\}$ (for measurement of x-direction momentum and y-axis position) and $C_{2}=\left\{X,P_{y}\right\}$ (for measurement of x-axis position and y-direction momentum) will each yield a well-defined value (and, hence, expectation and variance statistics). On the other hand, x-axis position and x-direction momentum *cannot be* jointly sharply determined—indeed, there is no sharp joint observable for their product; the product of their Hermitian operators, $XP_{X}$, is not itself Hermitian. Concomitantly, there exists no quantum context, in our sense, for the simultaneous measurement of the corresponding sharp observables. One sees that the measurement of these quantities is contextual in the above sense.

The HPI in the realm of generalized observables implies a more non-trivial contextuality, in that simultaneously measurements of the same quantities in different contexts can have different but existent statistics, in particular, the standard deviation of values for two quantities, A and B, across contexts involving the measurement of coexistent unsharp observables of properties, the sharp observables for which are *incompatible*.

In the general quantum setting, each context is identified by the circumstances of measurement, such as the coupling strengths of detection systems for the observables involved, for example, in conformance with the Arthurs–Kelly model (cf. Reference [8], Section II.2.3, pp. 152–153). For position and momentum, these are reflected in the (coexistent) unsharp observables $E^{e}$ and $F^{f}$ generalizing the projection-valued measures *E* and *F*, obtained by the convolution of *E* and *F* with (confidence) functions *e* and *f*—for example, corresponding to couplings and providing weights over *E* and *F* respectively (see Equations (10) and (11) below)—for the (incompatible) sharp measurements of position and momentum (here considered in one dimension); [8], pp. 59–65. For the corresponding indeterminacy relations to hold, it is sufficient for the unsharp observables $E^{e}$ and $F^{f}$ to be a Fourier couple. In particular, the confidence functions e,f are taken such that Δ(e)Δ(f)≥1/2; cf. Reference [8], p. 108, Reference [28]. This (Fourier) dispersion property of the confidence functions is a basis for the pair $E^{e},F^{f}$ to be referred to as a “Fourier couple.” (An extended exposition of the mathematical properties of Fourier couples, their dispersion, appearance in the theory of joint distributions initiated by Wigner and Gabor, and other applications can be found in, for example, Reference [28]. Here, the (bounded) confidence functions are of the form $e\left(q\right)=S_{0}\left(q,q\right),f\left(p\right)={\hat{S}}_{0}\left(p,p\right)$, where “^” indicates the momentum representation, and $S_{0}\left(\cdot ,\cdot \right)$ is the matrix element of the phase space density $S_{0}$, which is a positive trace-class operator of unit trace.)

The functions e(q),f(p) provide weights over the family of associated projections E,F appearing in the convolutions over position and momentum associated with the phase space, yielding the unsharp observables:

(10) $$E^{e}\left(x\right)=\int e\left(q\right)E\left(x+q\right)dq$$

and

(11) $$F^{f}\left(y\right)=\int f\left(p\right)F\left(y+p\right)dp.$$

Each pair of such unsharp observables $E^{e},F^{f}$ then provides a context $C_{e,f}=\left\{E^{e},F^{f}\right\}$ corresponding to the joint determination of unsharp position and momentum by an equivalence class of measurement arrangements. Taking confidence functions with vanishing first and finite second moments, one has

(12) $$\int qdE^{e}\left(q\right)=\int qdE\left(q\right)=X$$

(13) $$\int pdF^{f}\left(p\right)=\int pdF\left(p\right)=P$$

and

(14) $$\mathrm{Var}\left(E^{e},\psi \right)=\mathrm{Var}\left(E,\psi \right)+\mathrm{Var}\left(e\right)$$

(15) $$\mathrm{Var}\left(F^{f},\psi \right)=\mathrm{Var}\left(F,\psi \right)+\mathrm{Var}\left(f\right),$$

where ψ is the (pure) system state and $\mathrm{Var}(O^{o},\psi )$ indicates the variance of the probability measure $p_{\psi }^{O^{o}}$ for its observable *O* and confidence function *o* [8], p. 60. The following theorem (Busch et al. [8], p. 63) then expresses the HPI for unsharp position and momentum that implies contextuality for any quantum state *T*.

A pair $E^{e},F^{f}$ of unsharp position and momentum observables are jointly measurable (by means of a continuous phase space observable) if they are a Fourier couple, where *e* and *f* are confidence functions. In this case the standard deviations of $E^{e}$ and $F^{f}$ satisfy the uncertainty relation

(16) $$\mathrm{Var}\left(E^{e},T\right)\mathrm{Var}\left(F^{f},T\right)\geq 1,$$

for any quantum state *T*.

One sees that there can be different deviation statistics and, so, contextuality for the position and momentum in any state *T* depending on the nature of the apparatus and its couplings for the joint measurements simultaneously performed, that is, the context, the entire range of possible values of which can be taken as expressed by e,f: $E^{e}$ and $F^{f}$ have, in general, differing standard deviations across the set of contexts $\left\{C_{e,f}\right\}$. Most evidently, consider a pair of confidence functions $e_{0},f_{0}$ for which the inequality of Equation (16) is saturated and another pair, e,f, for which it is not; then, either $\mathrm{Var}\left(E^{e},T\right)\neq \mathrm{Var}\left(E^{e_{0}},T\right)$, $\mathrm{Var}\left(F^{f},T\right)\neq \mathrm{Var}\left(F^{f_{0}},T\right)$, or both, exhibiting a difference of variance statistics. Thus, contextuality is implied directly by the HPI.

## 5. Conclusions

In quantitative form, Heisenberg’s Principle of Indeterminism is expressible via a product of variances, that is, indeterminacy relations. Here, the HPI is shown to be sufficient for the existence of natural quantum contextuality in quantum mechanics in the setting of generalized observables. This is done by demonstrating the dependence of position and momentum statistics on measurement context using the HPI for simultaneously measurable, coexistent unsharp-observable counterparts of those observables considered in early stages of the theory’s development, when the HPI was first stated. A quantitative form of contextuality is thereby seen to be an inherent feature of quantum mechanics, rather than one imported via some alternative mechanical model or purely philosophical notion.

## References

[1] Jaeger G. (2019). Quantum Contextuality in the Copenhagen Approach. Philos. Trans. R. Soc. A.

[2] Haven E., Khrennikov A. (2013). Quantum Social Science.

[3] Cervantes V.H., Dzhafarov E.N. (2018). Snow Queen is Evil and Beautiful: Experimental evidence for probabilistic contextuality in human choices. Decision.

[4] Cabello A. (2008). Experimentally Testable State-Independent Quantum Contextuality. Phys. Rev. Lett..

[5] Kitto K., Ramm B., Sitbon L. (2011). Quantum Theory Beyond the Physical: Information in Context. Axiomathes.

[6] Busch P., Grabowski M., Lahti P. (1996). The Quantum Theory of Measurement.

[7] Busch P., Grabowski M., Lahti P. (1995). Repeatable Measurements in Quantum Theory: Their role and Feasibility. Found. Phys..

[8] Busch P., Grabowski M., Lahti P. (1997). Operational Quantum Physics.

[9] Busch P., Jaeger G. (2010). Unsharp Quantum Reality. Found. Phys..

[10] Heisenberg W. (1930). The Physical Principles of Quantum Theory.

[11] Jaeger G. (2017). Quantum randomness and Unpredictability. Fortschr. Phys..

[12] Heisenberg W., Price W.C., Chissick S.S. (1977). Remarks on the Origin of the Relations of Uncertainty. The Uncertainty Principle and Foundations of Quantum Mechanics: A Fifty-Year Survey.

[13] Heisenberg W. (1927). Über den anschaulichen Inhalt der quantentheoretischen Kinematik und Mechanik. Z. Phys..

[14] Arnold V.I. (1990). Huygens and Barrow, Newton and Hooke.

[15] Price W.C., Chissick S.S. (1977). The Uncertainty Principle and Foundations of Quantum Mechanics: A Fifty-Year Survey.

[16] Beller M. (1999). Quantum Dialogue: The Making of a Revolution.

[17] Hilgevoord J., Uffink J., Zalta E.N. (2016). The Uncertainty Principle. The Stanford Encyclopedia of Philosophy.

[18] Dirac P.A.M. (1930). The Principles of Quantum Mechanics.

[19] Von Neumann J. (1932). Mathematische Grundlagen der Quantenmechanik.

[20] Von Neumann J. (1955). Mathematical Foundations of Quantum Mechanics.

[21] Beyer R.T. (1932). Mathematische Grundlagen der Quantenmechanik.

[22] Robertson H.P. (1929). The Uncertainty Principle. Phys. Rev..

[23] Gudder S., Price W.C., Chissick S.S. (1977). Four Approaches to Axiomatic Quantum Mechanics. The Uncertainty Principle and Foundations of Quantum Mechanics: A Fifty-Year Survey.

[24] Sakurai J.J. (1994). Modern Quantum Mechanics.

[25] Ozawa M. (2003). Universally valid reformulation of the Heisenberg uncertainty principle on noise and disturbance in measurement. Phys. Rev. A.

[26] Bohr N. (1938). Natural Philosophy and Human Cultures. Nature.

[27] Jaeger G. (2016). Grounding the Randomness of Quantum Measurement. Philos. Trans. R. Soc. A.

[28] Roy S., Kundu M.K., Granlund G.H. (1996). Uncertainty Relations and Time-Frequency Distributions for Unsharp Observables. Inf. Sci..

